# Greater Impact of Living Donation Than HLA Mismatching in Short-Term Renal Allograft Survival

**DOI:** 10.7759/cureus.34427

**Published:** 2023-01-31

**Authors:** Bárbara Ribeiro, Pedro Reis Pereira, João Oliveira, Manuela Almeida, La Salete Martins, Jorge Malheiro

**Affiliations:** 1 Nephrology Department, Hospital de Braga, Braga, PRT; 2 Nephrology, Dialysis and Transplantation, Unit for Multidisciplinary Research in Biomedicine, Instituto de Ciências Biomédicas Abel Salazar (ICBAS), Porto, PRT; 3 Nephrology, Laboratory for Integrative and Translational Research in Population Health (ITR), Porto, PRT; 4 Nephrology, Centro Hospitalar de Trás-os-Montes e Alto Douro, Vila Real, PRT; 5 Nephrology, Centro Hospitalar Universitário do Porto, Porto, PRT

**Keywords:** global graft survival, censored graft survival, acute rejection, hla mismatching, deceased donor kidney transplantation, living donor kidney transplantation

## Abstract

Introduction: Living donor kidney transplantation (LDKT) is accepted as first-line treatment for patients with end-stage kidney disease with advantages over deceased donor kidney transplantation (DDKT). Still, how the known detrimental effect of HLA mismatch (MM) may hamper these advantages remains unsettled. We sought to determine the effect of the degree of HLA MM, separately in deceased and living donor renal allograft outcomes.

Methods: We evaluated all adults submitted to LDKT and DDKT at our center between 2006 and 2018. Their HLA MM was classified according to the British Society of Transplantation system in low mismatch (LM) (level 1-2) and high mismatch (HM) (level 3-4). Acute rejection (AR) and global or censored graft survival were the outcomes of interest. Recipients were followed up from transplant until death, graft failure or the end of 2020.

Results: One thousand sixty-eight kidney transplant recipients were analyzed, 815 (76%) received a DDKT whereas 253 (24%) received an LDKT. From those submitted to DDKT, 95 (12%) had an LM and 720 (88%) had an HM, whereas in LDKT 32 (13%) had an LM and 221 (87%) had an HM. The AR at one year was 9% in the full cohort. Significant risk factors for AR were HM DDKT (OR:2.3, P=0.047) or HM LDKT (OR:5.6, P=0.003) (LM DDKT as reference), calculated panel-reactive antibody (cPRA) ≥5% (OR:1.9, P=0.040) and delayed graft function (DGF), (OR:3.2, P<0.001). Censored graft survival (CGS) at five years was 96% in the full cohort. Independent predictors for censored graft failure (CGF) were HM LDKT (HR:0.2, P=0.046) (LM DDKT as reference), AR (HR:2.7, P=0.008) and DGF (HR:2.2, P=0.017). Global graft survival (GGS) at five years was 91% in the full cohort. Independent predictors for global graft failure (GGF) were HM LDKT (HR:0.2, P=0.042) (LM DDKT as reference), recipient age (HR:1.8, P<0.001) and DGF (HR:1.8, P=0.006). No AR, CGF or GGF episodes were observed in the LM LDKT group.

Conclusions: In our cohort, the level of HLA MM increased the risk of AR independently of donor type. Considering short graft survival, our results support the advantage of living donor vs deceased donor even with an increased HLA MM. However, its effect on long-term graft survival remains to be settled, emphasizing the need for further studies on this matter.

## Introduction

Living donor kidney transplant (LDKT) is the best treatment for suitable patients with end-stage renal disease. It offers several advantages as it allows a shorter waiting time with the possibility of transplant before dialysis (preemptive), a lower rate of delayed graft function and improved long-term graft survival, even from genetically unrelated donors, over those who receive deceased donor grafts [1,2]. The scarcity of organs, despite the good policy of deceased organ retrieval, is a critical problem. Opting for living donation is one of the possibilities to increase organ supply, particularly from non-related donation and exchange programs [3]. The expansion of LDKT programs over the last decades was only possible because of the growth of living unrelated kidney donation (LURD), largely associated with a declining emphasis on close human leukocyte antigen (HLA) matches between donor-recipient pairs [4,5]. HLA matching is reported to have a less significant effect on short-term kidney allograft outcomes than previously thought, but the long-term impact is still unclear [6]. The current estimated lifetime of a transplant kidney is 10-15 years, therefore young recipients should be expected to undergo more than one kidney transplantation during their lifetime [7]. The impact of poor HLA matching at the first transplant can translate into a more complicated future transplantation due to HLA sensitization [8,9]. For instance, the hypersensitized population of kidney transplant candidates in Portugal is increasing, with the national registry of 2020 showing a prevalence of 31.3% of patients in the waiting list with calculated panel reactive antibodies (cPRA) over 98% (according to the loci HLA-ABDR) [10,11]. So, it is time to reassess the effects of HLA mismatch (MM) in long-term graft and patient survival in living donor kidney recipients. The aim of this study was to determine the effect of the degree of HLA mismatching separately in deceased and living donor renal allograft outcomes in adult recipients.

## Materials and methods

Population and study cohort

We retrospectively evaluated all adults submitted to LDKT and deceased-donor kidney transplant (DDKT) at our institution between 2006 and 2018 (n=1193). After the exclusion of 63 patients whose degree of mismatching was unknown, 43 patients who had a primary loss of KT and 19 HLA incompatible transplants, the remaining 1068 recipients defined our study cohort.

HLA matching classification

The HLA mismatch was classified according to the British Society of Transplantation system as low mismatch (LM) (level 1-2) and high mismatch (HM) (level 3-4) [7]. The four possible levels of mismatch were grouped and analyzed in two classifications: a high HLA match (level 1 or level 2) and a low HLA match (level 3 or level 4) (Table 1).

**Table 1:** The proportion of patients with each level and group of HLA matching for each transplant type (DD and LD) HLA: human leukocyte antigen, DD: deceased donor, LD: living donor, L: level, LM: low mismatch, HM: high mismatch

HLA match level	DD	LD	Total
L1, n (%)	8 (1)	17 (7)	25
L2, n (%)	87 (11)	15 (6)	102
L3, n (%)	285 (35)	101 (40)	386
L4, n (%)	435 (53)	120 (47)	555
LM (L1+L2), n (%)	95 (12)	32 (13)	127
HM (L3+L4), n (%)	720 (88)	221 (87)	941
Total	815 (100)	253 (100)	1068

Baseline data and graft outcomes

Baseline demographic and clinical data were collected from both recipients and donors. Transplant data were also collected and analyzed. Delayed graft function (DGF) was assumed when there was need for dialytic support during the first post transplantation week. Graft biopsies were performed by indication. All acute rejection (AR) episodes were supported with biopsy, classified according to Banff’17 criteria and recorded during the first year after procedure. Recipients were followed up from transplant until death, graft failure or the end of 2020. The study protocol was reviewed and approved by the institutional ethical review and hospital administration boards of Centro Hospitalar Universitário do Porto and Instituto de Ciências Biomédicas Abel Salazar (approval no. 159/21) as per the recommendations of the Declaration of Helsinki and European Data Protection Regulations.

Immunosuppression (IS) and desensitization protocols

Induction therapy with an anti-interleukin (IL)-2 receptor monoclonal antibody (basiliximab 20 mg twice on days 0 and four) or with polyclonal anti-thymocyte globulin (ATG) (3 mg/kg for five to seven days) was used in most patients. Anti-thymocyte globulin was used primarily in patients with cPRA >60% and in patients submitted to retransplantation. The great majority of recipients (>90%) received triple maintenance immunosuppression with oral tacrolimus, mycophenolate mofetil (MMF) and corticosteroids. Tacrolimus was started at a dose of 0.1-0.15 mg/kg/day, which was then adjusted to maintain a trough level between 8 and 12 ng/ml during the first post transplantation month, between 7 and 10 ng/ml after two to three months and between 5 and 8 ng/ml long-term. MMF was initiated at a dose of 2000 mg/day, with decreasing dose to 1000-1500 mg/day during the first month postoperatively, adjusted to white blood cells count. Both tacrolimus and MMF were started one week before transplant in LDKT recipients. Methylprednisolone was administered at doses of 500 mg on the transplantation day, 250 mg the two days after and 125 mg on day three and four after the operation. Oral prednisolone was then at the dose of 20 mg and tapered to 5-10 mg/day within two to three months.

Statistical analysis

Continuous data are described as mean ± standard deviation (SD) or median (interquartile range [IQR]). Categorical data are expressed in numbers and percentages. Categorical data were compared using Pearson’s chi-square test or Fisher’s exact test, and continuous variables were compared using Student’s t-test or Mann-Whitney U-test.

AR and graft survival curves were visualized using the Kaplan-Meier method, with a comparison between patients' groups done by log-rank test. In the case of death with a functioning graft, time was censored at the time of death. Potential predictors of AR and censored and global graft failure were explored by univariate and multivariable Cox proportional hazards models. In all multivariable models, independent predictors were identified using a backward elimination method, with a P-value < 0.05 necessary for retention in the model. A 2-sided P-value < 0.05 was considered as statistically significant. Statistical calculations were performed using STATA/MP, version 15.1 (Stata Corp., College Station, TX, USA).

## Results

Characteristics of patients

Our study cohort comprised 1068 recipients; 815 (76%) received a DDKT whereas 253 (24%) received an LDKT. From those submitted to DDKT, 95 (12%) had an LM and 720 (88%) had an HM, whereas in LDKT 32 (13%) had an LM and 221 (87%) had an HM. Table 2 describes the baseline characteristics for all recipients, donors, transplant characteristics and immunosuppressive treatment specificities included in the analysis, divided by HLA match group and transplant type.

**Table 2:** Baseline characteristics of living and deceased donor and transplants. R: recipient; SD: standard deviation; F: female; KT: kidney transplant; IQR: interquartile range; RRT: renal replacement therapy; D: donor; DD: deceased donor; LD: living donor; PRA: panel-reactive antibodies; HLA: human leukocyte antigen; MM: mismatch; LM: low mismatch; HM: high mismatch; ATG: antithymocyte globulin; TAC: tacrolimus; CSA: ciclosporine; MMF: mofetil mycophenolate; pred: prednisone; DGF: delayed graft function.

	Total N=1068	DD LM N=95 (9%)	DD HM N=720 (67%)	LD LM N=32 (3%)	LD HM N=221 (21%)	P-value
Recipient						
Age of R, mean ±SD	46.8±14.9	50.1±13.7	48.4±14.8	41.0±11.5	40.8±14.1	<0.001
Age of R ≥50, n (%)	487 (46)	51 (54)	366 (51)	6 (19)	64 (29)	<0.001
Sex of R (F), n (%)	399 (37)	37 (39)	281 (39)	12 (38)	69 (31)	0.211
Time on dialysis before KT (years), n (%)						<0.001
0-1	172 (16)	8 (8)	53 (7)	14 (44)	97 (44)	
1-3	210 (20)	22 (23)	102 (14)	8 (25)	78 (35)	
3-5	245 (23)	28 (29)	197 (27)	2 (6)	18 (8)	
≥5	441 (41)	37 (39)	368 (51)	8 (25)	28 (13)	
Donor						
Age of D, mean ±SD Missing: 98	48.5±14.3	49.0±13.5	49.0±15.5	49.4±11.6	46.7±10.3	0.243
Age of D ≥50, n (%)	499 (51)	45 (55)	356 (54)	16 (57)	82 (42)	0.021
Sex of D (F), n (%)	372 (39)	24 (30)	180 (28)	20 (63)	148 (71)	<0.001
RRT pre-KT, n (%)						
Preemptive	67 (6)	1 (1)	10 (1)	6 (19)	50 (23)	<0.001
Transplant						
Year of KT, n (%)						0.006
2006-2010	377 (35)	44 (46)	263 (37)	8 (25)	62 (28)	
2011-2014	323 (30)	30 (32)	219 (30)	9 (28)	65 (29)	
2015-2018	368 (34)	21 (22)	238 (33)	15 (47)	94 (43)	
Retransplant, n (%)	155 (15)	10 (11)	108 (15)	9 (28)	28 (13)	0.081
Calculated PRA >5%, n (%)	131 (13)	12 (15)	103 (16)	2 (7)	14 (7)	0.011
HLA-ABDR MM, mean±SD	3.86 (0.04)	2.01 (0.09)	4.22 (0.04)	0.72 (0.16)	3.91 (0.08)	<0.001
HLA-A MM, mean±SD	1.33 (0.02)	1.25 (0.06)	1.42 (0.02)	0.47 (0.10)	1.18 (0.04)	<0.001
HLA-B MM, mean±SD	1.40 (0.02)	0.77 (0.04)	1.52 (0.02)	0.25 (0.08)	1.44 (0.04)	<0.001
HLA-DR MM, mean±SD	1.13 (0.02)	0 (0)	1.28 (0.02)	0 (0)	1.29 (0.03)	<0.001
IS Induction, n (%)						<0.001
Without	174 (16)	24 (25)	138 (19)	5 (16)	7 (3)	
Basiliximab	683 (64)	56 (59)	405 (56)	25 (78)	197 (89)	
ATG	211 (20)	15 (16)	177 (25)	2 (6)	17 (8)	
Maintenance IS, n (%)						0.004
TAC + MMF+ pred	1013 (95)	87 (92)	689 (96)	31 (97)	206 (93)	
CSA + MMF + pred	28 (3)	2 (2)	12 (2)	1 (3)	13 (6)	
Others	27 (3)	6 (6)	19 (3)	0	2 (1)	
DGF, n (%)	181 (17)	22 (23)	153 (21)	2 (6)	4 (2)	<0.001
Follow-up (years), median (IQR)	5 (4.0-5)	5 (5-5)	5 (3.8-5)	5 (4.4-5)	5 (4.2-5)	0.094

The mean recipient age at the time of kidney transplantation was lower in LDKT groups compared to DDKT groups (P<0.001). Fifteen percent of all patients had a previous transplant and the percentage of preemptive kidney transplants was superior in LDKT groups (19% with LM and 23% with HM) compared to DDKT groups (only 1%, P<0.001).

ATG was used as an induction in 20% of the cohort, particularly in HM DDKT (25%). DGF occurred in 20% of all cohort and was more frequent on DDKT, regardless of HLA mismatch level.

Acute rejection analysis by HLA match group

Acute rejection (AR) at one year was 9% in the full cohort and was superior in HM DDKT and HM LDKT groups, 10% and 12%, respectively (P=0.039), as shown in Table 3. In the multivariate analysis, HM DDKT (OR:2.3, P=0.047), HM LDKT (OR:5.6, P=0.003) (LM DDKT as reference), cPRA ≥5% (OR:1.9, P=0.040) and delayed graft function (DGF, OR:3.2, P<0.001) were significant risk factors for AR (after adjustment for the recipient and donor age, sex, immunosuppression induction regimen, immunosuppression manutention, re-transplantation rate and previous time on kidney replacement therapy) (Table 4). In contrast, kidney transplantation occurring in the 2015-2018 timeframe (2006-2010 as a reference) was associated with a significantly lower risk of AR (HR=0.470, P=0.003). No AR episode was observed in the LM LDKT group.

**Table 3:** Outcomes of interest DD: deceased donor, LD: living donor, LM: low mismatch, HM: high mismatch.

	Total N=1068	DD LM N=95 (9%)	DD HM N=720 (67%)	LD LM N=32 (3%)	LD HM N=221 (21%)	P-value
Acute rejection at 1-year, n (%)	99 (9)	4 (4)	69 (10)	0	26 (12)	0.039
Censured graft failure at 5-year, n (%)	43 (4)	5 (5)	36 (5)	0	2 (1)	0.016
Global graft failure at 5-year, n (%)	96 (9)	8 (8)	86 (12)	0	2 (1)	<0.001
Deaths at 5-year, n (%)	53 (5)	3 (3)	50 (7)	0	0	<0.001

**Table 4:** Predictors of acute rejection Adjusted to recipient age, donor age, recipient sex, donor sex, induction IS, maintenance IS, retransplant rate, months on RRT, time period of KT and cPRA. CI: confidence interval; cPRA: calculated panel-reactive antibody; DD: deceased donor; KT: kidney transplant; LD: living donor; LM: low mismatch; HM: high mismatch; IS: immunosuppression; RRT: renal replacement therapy.

	HR (CI 95%)	P-value
HLA/donor		
LM DD	1 (Reference)	
HM DD	2.298 (1.014-8.458)	0.164
LM LD	- (no events)	-
HM LD	5.600 (1.773-17.687)	0.042
Year of transplant		
2006-2010	1 (Reference)	
2011-2014	0.568 (0.352-0.916)	0.020
2015-2018	0.500 (0.297-0.841)	0.009
Delayed graft function	3.174 (1.932-5.216)	<0.001
cPRA >5%	1.852 (1.027-3.341)	0.040

Allograft and patient survival analysis by HLA match group

**Figure 1:**
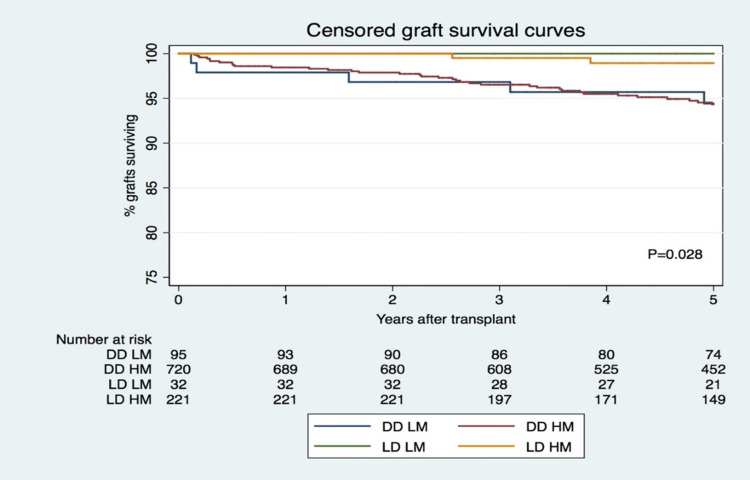
Censored graft survival DD: deceased donor; LD: living donor; LM: low mismatch; HM: high mismatch.

**Figure 2:**
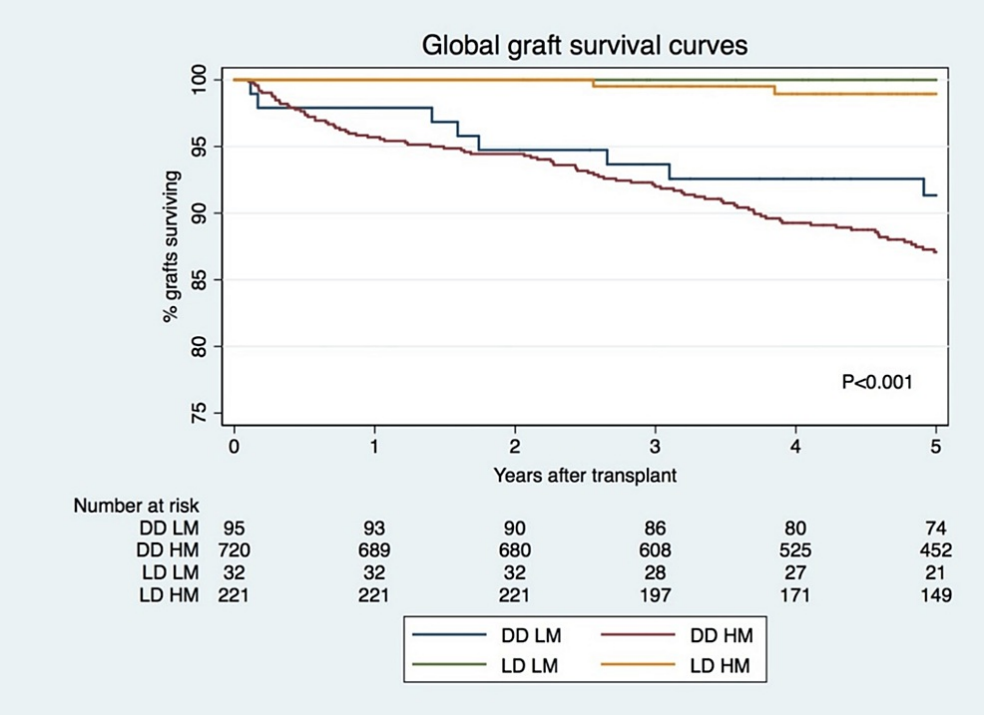
Global graft survival DD: deceased donor; LD: living donor; LM: low mismatch; HM: high mismatch.

Five-year censored and global renal allograft survivals by donor type and HLA match group are shown in Figure 1 and Figure 2, respectively. This shows renal allograft survival results after well and poorly HLA-matched DDKT and survival after well and poorly HLA-matched LDKT. Five-year censored renal allograft survival was shown to be superior for patients receiving a poorly HLA-matched LDKT at 98.8% compared with those receiving a well HLA-matched DDKT, which was 94.3% (P=0.028). For both forms of donor graft, there was no statistical evidence to suggest a significant difference in renal allograft survival in patients who received a good HLA match compared with those that received a poor HLA match. Similar results were observed for global graft survival at five years. Global graft survival at five years was superior for HM LDKT (98.9%) compared to LM DDKT (91.3%). Censored and global graft failure at five years was significantly superior in groups of DDKT, as shown in Table 3. Independent predictors for censured graft failure (CGF) at five years were HM LDKT (HR:0.2, P=0.046) (LM DDKT as reference), AR (HR:2.7, P=0.008) and DGF (HR:2.2, P=0.017). No CGF episode was observed in the LM LDKT group (Table 5). Independent predictors for global graft failure (GGF) were HM LDKT (HR:0.2, P=0.042) (LM DDKT as reference), recipient age (HR:1.8, P<0.001) and delayed graft function (DGF, HR:1.8, P=0.006) (Table 6). No GGF episode was observed in the LM LDKT group. Patient survival was 100% in LDKT groups and a significant mortality rate was observed in the HM DDKT group (n=50, 7%) and in the LM DDKT (n=3, 3%) (P<0.001) (Table 3).

**Table 5:** Predictors of censored graft failure Adjusted to recipient age, donor age, recipient sex, donor sex, induction IS, maintenance IS, retransplant rate, months on RRT, time period of KT and cPRA. CI: confidence interval; cPRA: calculated panel-reactive antibody; DD: deceased donor; KT: kidney transplant; LD: living donor; LM: low mismatch; HM: high mismatch; IS: immunosuppression; RRT: renal replacement therapy.

	HR (CI 95%)	P-value
HLA/donor		
LM DD	1	
HM DD	0.953 (0.372-2.444)	0.920
LM LD	- (no events)	-
HM LD	0.182 (0.034-0.969)	0.046
Acute rejection	2.661 (1.291-5.487)	0.008
Delayed graft function	2.207 (1.151-4.232)	0.017

**Table 6:** Predictors of global graft failure Adjusted to recipient age, donor age, recipient sex, donor sex, induction IS, maintenance IS, retransplant rate, months on RRT, time period of KT and cPRA. CI: confidence interval; cPRA: calculated panel-reactive antibody; DD: deceased donor; KT: kidney transplant; LD: living donor; LM: low mismatch; HM: high mismatch; IS immunosuppression; RRT: renal replacement therapy.

	HR (CI 95%)	P
HLA/donor		
LM DD	1	
HM DD	1.675 (0.810-3.462)	0.164
LM LD	- (no events)	-
HM LD	0.197 (0.041-0.942)	0.042
Recipient age	2.661 (1.291-5.487)	0.008
Delayed graft function	2.207 (1.151-4.232)	0.017
Year of transplant		
2006-2010	1	
2011-2014	0.568 (0.352-0.916)	0.020
2015-2018	0.500 (0.297-0.841)	0.009

## Discussion

As documented in the available literature, renal allograft survival is improved for recipients of living donors compared with deceased donors [12,13]. Additionally, LDKT can also help to decrease organ scarcity and waitlists [14]. On the other hand, in opposition to time on dialysis, HLA matching is not a priority in our national allocation system and consequently the hypersensitized transplant population is increasing in the last years [10,15]. The number of HLA MM is associated with an increased risk of poorer deceased donor transplant outcomes and de novo post-transplant donor-specific antibodies (DSA) due to HLA MM are associated with graft failure [16-18]. However, the role of HLA MM in living donor allograft survival is not clearly defined.

There are reports suggesting that HLA matching may not be as important in LDKT as it is in DDKT. A retrospective analysis of Casey et al. matched 83 0-HLA MM to 407 controls with ≥1 HLA MM in living donors. The authors reported that 0-HLA MM did not confer improved death-censored graft and patient survival [19]. In another study, while HLA-DR MM was a risk factor for acute rejection, HLA MM did not increase the risk of allograft loss or patient death [20].

In contrast, an analysis based on the data of the International Collaborative Transplant Study showed a strong and statistically significant impact of HLA-A+B+DR MMs on the survival of pediatric transplants from living donors [21]. Furthermore, they showed significantly better transplant outcomes with kidneys from deceased donors with 0 to one HLA MMs as compared to transplants from living donors with four to six MMs, concluding that LDKT is an excellent option, since the number of HLA-A+B+DR MMs does not exceed three [21]. Additionally, a recent analysis of United States registry data on HLA matching in LDKT observed an inverse correlation between allograft survival and number of HLA MM. They also showed that any living donor kidney with two MM is significantly better than a kidney from a deceased donor with 0 MM. However, when there are three MM, the HRs for living allografts are not significantly different from the DD with 0 HLA MM [22].

Our study design was similar to the one conducted by Marlais et al. that addressed the effect of HLA matching on deceased and living donor renal allograft outcomes in pediatric recipients. They tried to understand whether it is better to wait for a closely matched organ from a deceased donor or to decide to provide an organ from a living donor with HM [7]. The results demonstrated that children receiving a poorly HLA-matched LDKT do not have an inferior five-year renal allograft survival compared to those receiving a well HLA-matched DDKT, making it hard to justify the wait [7]. 

Our results also showed that the censored and global allograft survivals were significantly better in the HM LDKT group compared to the LM DDKT group. Furthermore, the patients who received a poorly matched LD kidney transplant have an 82% lower risk of censored graft failure and an 80% lower risk of global graft failure, supporting the benefits of LDKT above DDKT. However, it is still early to try to answer if it is worth the wait for LM DDKT or if it is better to proceed to HM LDKT, also due to the lack of data on the impact of time of the waiting list in patient survival [23].

In our study, HLA MM increased the risk of AR, independently of donor type with greater impact on the HM LDKT group. This finding raises concern about the potential immunosuppression risks in the short and long term, particularly infections and neoplasms, and stresses the problem of the increasing number of hypersensitive patients on the waiting list. This could be possibly overcome through the inclusion of compatible pairs in kidney-paired exchange programs, with the goal of obtaining a better HLA or eplets match. The pairing of donor and recipients through these programs increases the transplant opportunities for all patients on the waitlist and would favor highly sensitized and blood O type recipients [3,23,24]. Decreasing HLA and eplets MM, especially in class II, could improve significantly long-term survival of renal grafts as the degree of MM is associated with increased rejection rate [25].

The present study has several limitations. Firstly, it is unicentric and restricted to a five-year outcome timeframe on renal allografts, leading to the lack of assessment of the long-term effect of poorly HLA-matched transplantation. Secondly, it involved a prolonged recruitment time, which implicates both different immunosuppression and histocompatibility-testing protocols. Lastly, the population analyzed was exclusively Caucasian.

## Conclusions

In our cohort, the level of HLA mismatching increased the risk of AR, independently of donor type. Considering short graft survival, our results support the advantage of living donor vs deceased donor even with an increased HLA mismatch. However, its effect on long-term graft survival remains to be settled, emphasizing the need for further studies on this matter. Moreover, the absence of any outcomes of interest in the LM LDKT highlights the optimal choice represented by a low mismatch transplant, which in the case of LDKT could be reached by the introduction of compatible pairs in the kidney paired donation program.
